# 

**Published:** 1911-09

**Authors:** 


### EIGHTY-FIRST LIST OF BOOKS.

The titles of books mentioned in previous lists are not repeated.

The figures in brackets refer to the figures after the names of the donors, and show by whom the volumes were presented. The books to which no such figures are attached have either been bought from the Library Fund, or received through the *Journal.*

**Bandelier and Roepke** *Tuberculin in Diagnosis and Treatment* (Trans. by E. C. Morland) .. .. .. .. .. 1909

**Bass, G. Dock and C. C.** *Hookworm Disease* .. .. .. .. .. .. 1910

**Berdoe, E.** .. .. *The Origin and Growth of the Healing Art* .. 1893

**Box, C. R.** .. .. *Post-mortem Manual* .. .. .. .. .. .. 1910

**Dock and C. C. Bass, G.** *Hookworm Disease* .. .. .. .. .. .. 1910

**Eden, T. W.** .. *A Manual of Gynæcology* .. .. .. .. .. 1911

**Gee, S.** .. .. .. *Medical Lectures and Aphorisms* .. .. .. .. 1908

**Hare, H. A.** .. .. *Modern Treatment.* 2 vols. .. .. .. .. .. 1911

**Hartley, Sir R. D. Powell and P. H.-S.** *On Diseases of the Lungs and Pleuræ.* .. .. .. .. .. .. 5th Ed. 1911

**Hirschfelder, A. D.** *Diseases of the Heart and Aorta* .. .. .. [1910]

**Hutchinson, Sir J.** *The New Sydenham Society. Retrospective Memoranda* .. .. .. .. .. .. .. .. .. .. 1911

**Hutchinson, Sir F. Treves and J.** *Student's Handbook of Surgical Operations* .. .. .. .. .. .. 3rd Ed. 1911

**Love, J. K.** .. .. *The Deaf Child* .. .. .. .. .. .. .. .. .. .. 1911

**Melville-Davison, W.** *Ships' Hygiene* .. .. .. .. .. .. .. .. 1911

**Ochsner and R. L. Thompson, A. J.** *The Surgery and Pathology of Thyroid and Parathyroid Glands* .. .. .. 1910

**Powell and P. H.-S. Hartley, Sir R. D.** *On Diseases of the Lungs and Pleuræ* .. .. .. .. .. .. 5th Ed. 1911

**Ricketts, H. T.** .. *Contributions to Medical Science* .. .. .. [1911]

**Roepke, Bandelier and** *Tuberculin in Diagnosis and Treatment* (Trans. by E. C. Morland) .. .. .. .. .. 1909

**Russell, P. Sargent and A. E.** *Emergencies of General Practice* 2nd Ed. [1911]

**Sargent and A. E. Russell, P.** *Emergencies of General Practice* 2nd Ed. [1911]

**Smith, J. S. K.** *Lateral Curvature of the Spine and Flat-Foot* .. 1911

**Stewart, D.** .. .. *The Essentials of Food* .. .. .. .. .. .. .. 1911

**Thompson, A. J. Ochsner and R. L.** *The Surgery and Pathology of Thyroid and Parathyroid Glands* .. .. .. .. 1910

**Treves and J. Hutchinson, Sir F.** *Student's Handbook of Surgical Operations* .. .. .. .. .. .. 3rd Ed. 1911

**Waller, H. E.** .. *Theory and Practice of Thyroid Therapy* .. [1911]

### TRANSACTIONS, REPORTS, JOURNALS, &c.

College of Physicians of Philadelphia, Transactions of the Vol. XXXII 1910

Hospitals—

St. Thomas's Hospital Reports .. .. .. Vol. XXXVIII 1911

Laboratory Reports of the Royal College of Physicians of Edinburgh Vols. X, XI 1911

Medical Libraries .. .. .. .. .. .. .. .. Vols. III—V 1901–02

Progressive Medicine .. .. .. .. .. .. .. Vol. I 1911

Zeitschrift für Urologie .. .. .. .. .. .. .. .. .. Bd. IV 1910

### Obituary.

### University of Bristol.

RECENT EXAMINATION RESULTS.

UNIVERSITY OF LONDON.—*M.D. (Midwifery and Diseases of Women) :* R. E. Thomas, B.S.

*M.S. :* Cecil A. Joll, M.B., B.Sc.

*Second Examination for Medical Degrees, Part II, M.B., B.S. :* H. J. D. Smythe, E. S. Abraham.

UNIVERSITY OF BRISTOL.—*Final Examination :* E. E. Davies, C. A. H. Gee, E. F. Thomas, A. J. Wigmore. *Part I :* M. C. Barber, E. V. Foss, A. G. Hieber, B. J. F. Jackson-Taylor, C. Wintle. *Part II :* S. H. Kingston. *M.B., Ch.B., Second Examination :* R. I. Dacre, C. J. D. May, A. G. Morris.

*B.D.S. :* W. J. Lennox.

M.R.C.S., L.R.C.P. LOND.—A. G. T. Fisher, H. H. Hiley, V. Pinnock. *Chemistry and Physics :* H. R. W. Husbands, D. Munro Smith. *Materia Medica :* G. S. Terry. *Midwifery :* E. S. Abraham.

L.D.S., R.C.S. ENG.—*Preliminary Science Examination—Physics :* G. M. Hick, A. H. Virgin.

APPOINTMENTS.

Robert Donaldson, M.A., M.B., Ch.B., F.R.C.S. Edin., D.T.M., late Demonstrator of Pathology in the University of Sheffield, has been appointed Demonstrator of Pathology.

A. G. T. Fisher, M.R.C.S., L.R.C.P., has been appointed Demonstrator of Anatomy, vice Dr. Annan resigned.

W. Odell, M.D., F.R.C.S., has been appointed Hon. Consulting Physician, Western Hospital for Consumption, Torquay.

W. H. Symons, M.D., D.P.H., has been re-appointed Medical Officer of Health for Bath.

E. G. Bunbury, M.R.C.S., L.R.C.P., has been appointed District Medical Officer of the Helston Board of Guardians.

Capt. V. B. Green-Armytage, M.B., I.M.S., has been appointed Resident Medical Officer to the Eden Hospital, Calcutta.

